# Effect of Annealing Heat Treatment on the Composition, Morphology, Structure and Mechanical Properties of the W-S-N Coatings

**DOI:** 10.3390/ma15124088

**Published:** 2022-06-09

**Authors:** Talha Bin Yaqub, Abbas Al-Rjoub, Hafiza Ayesha Khalid, Khurram Yaqoob, Filipe Fernandes, Albano Cavaleiro

**Affiliations:** 1 CEMMPRE-Centre for Mechanical Engineering Materials and Processes, University of Coimbra, Rua Luís Reis Santos, 3030-788 Coimbra, Portugal; abbas.al-rjoub@dem.uc.pt (A.A.-R.); h.ayesha.khalid123@gmail.com (H.A.K.); filipe.fernandes@dem.uc.pt (F.F.); albano.cavaleiro@dem.uc.pt (A.C.); 2 Laboratory of Tests, Wear and Materials, IPN-LED & MAT-Instituto Pedro Nunes, Rua Pedro Nunes, 3030-199 Coimbra, Portugal; 3 School of Chemical and Materials Engineering (SCME), National University of Sciences and Technology (NUST), H-12, Islamabad 44000, Pakistan; khurram.yaqoob@scme.nust.edu.pk; 4 ISEP—School of Engineering, Polytechnic of Porto, Rua Dr. Antonio Bernardino de Almeida 431, 4200-072 Porto, Portugal

**Keywords:** transition metal dichalcogenides, W-S-N coatings, annealing, crystal structure, hardness, dry lubricants

## Abstract

Alloyed-transition metal dichalcogenide (TMD) coatings have been under investigation as multi-environment lubricants for the past few decades. These coatings display very low coefficient of friction properties at elevated temperatures. Studies on the annealing of these low-friction coatings are missing in the literature. For the first time, in this study, the annealing of the W-S-N dry lubricant coatings was carried out to study its effects on the composition, morphology, crystal structure and hardness of the coatings. The W-S-N coatings were deposited by direct current (DC) reactive magnetron sputtering. The analysis was carried out for as-deposited, 200 °C and 400 °C annealed coatings. The as-deposited coatings have N content in the range of 0–25.5 at. %. The coatings are compact and the densification increased with the increase in N-alloying. All the coatings are crystalline except the highest N-alloyed coating which is X-ray amorphous. A maximum hardness of 8.0 GPa was measured for the coating alloyed with 23 at. % N. Annealing did not affect the composition and morphology of the coatings, while some variations were observed in their crystal structure and hardness. The maximum hardness increased from 8 GPa to 9.2 GPa after 400 °C annealing of the 23 at. % N-alloyed coating.

## 1. Introduction

To analyse the reasons behind the response of engineering materials at high temperatures, heat treatment procedures such as annealing have long been one of the fundamental methods [1,2]. In fact, annealing is not only limited to bulk components but also it contributes to a major fraction of research involved with thin films. Both industrial and lab-scale research investigations involving synthesis of various components and parts use annealing studies to better understand the response of high temperature materials [3,4]. These studies rendered fruitful results in unveiling many ambiguities faced during the analysis of the performance of materials at high temperatures. Recently, annealing studies have been widely conducted for dry lubricant thin coatings to understand the high temperature stability and oxidation resistance [5,6,7,8]. During high-temperature sliding of dry-lubricant coatings, there are still many unanswered questions related to the reasons behind low/high friction coefficient (COF), enhanced crystallisation, oxidation behaviour, diffusion of lubricant phase to the surface, the effect of temperatures on life cycle, etc. The answers can be predicted/understood by performing annealing studies of these coatings. Moreover, as each material/component has its specific application environment, annealing allows the freedom to understand these fluctuations in properties in desired atmospheres (such as dry N_2_, vacuum, ambient air, etc.).

Among the most famous dry lubricant coatings used in the industry, the transition metal dichalcogenides (TMDs) are well known for their applications in the aerospace industry [9,10,11,12,13]. TMDs show excellent sliding performance in a vacuum and poor performance in ambient atmospheres [14,15]. TMDs have been alloyed with different metals [16,17,18,19,20,21,22,23] and non-metals such as C and N [10,24,25,26,27,28,29,30,31,32], to improve their sliding efficiencies in diverse environments (ambient air at room temperature, ambient air at elevated temperatures, dry N_2_ and vacuum). Until now, the most successful approaches include the alloying of TMDs with non-metals (C and N). After these improved research results, their industrial applicability is expected to be enhanced.

Currently, the need for green dry-lubricant systems that can work in multiple conditions is growing continuously. So, reducing either the drawbacks of lubrication degradation with environmental shifts or the harmful environmental effects of liquid lubricants is essential. Considering the market, universal solid lubricants that can present low COF in diverse environments (especially for the aerospace industry) are demanded. In the recent past, our group has reported various studies on the lab-scale and semi-industrial scale optimisations of different TMDs alloyed with C and N [11,31,32,33,34]. During these studies, the sliding properties of these C- and N-alloyed coatings have been tested in ambient air at both room temperature and elevated temperatures. From these studies, we can infer that the frictional performance of the coatings improves with an increase in temperature, i.e., the COF at room temperature is higher than the one at elevated temperatures (obviously up to a certain limit in which the coating does not degrade). Similar results in room- and high-temperature sliding have also been reported by other research groups [29,35,36].

Out of the various subsets of the N- and C-alloyed TMD coatings, the W-S-C and W-S-N coatings remain mostly studied by the researchers. In particular, the W-S-C coatings received the highest attention, whilst the W-S-N coatings were superficially explored, especially with respect to high-temperature sliding performance. Recently, the present authors reported a detailed study on the synthesis, characterisation and tribological properties of W-S-N coatings [37]. In that study, the sliding properties of these W-S-N coatings were explored at elevated temperatures, a scarcely investigated area so far. In agreement with the literature on alloyed WS_2_ coatings, the COF decreases with an increase in the temperature.

Upon exploration of the previous literature, we realized that there are no studies that have reported the effects of annealing on the alloyed-TMD coatings. Similarly, there are no studies on the effects of protective atmosphere annealing on the composition, morphology, structure and hardness of W-S-N coatings. In our opinion, it is crucial to investigate this for a better understanding of the reasons behind the response of the coatings at high temperatures. Moreover, some industrial components coated with the TMD coatings are subjected to high temperatures and their performance needs to be understood. In this study, the effect of annealing treatment on the compositional, structural, morphological and hardness of W-S-N coatings in protective environment as a continuation of the previous study (see ref. [37]) is investigated to answer these questions.

## 2. Materials and Methods

### 2.1. Coating Synthesis

W-S-N coatings with varying N contents were deposited by direct-current reactive magnetron sputtering in a Hartec^®^ deposition chamber equipped with 2 cathodes (WS_2_ and Cr). The dimensions and purity of both targets are 150 mm × 150 mm × 8 mm and 99.99% purity, respectively. During the synthesis of the coatings, the power applied to the WS_2_ target was kept constant while the N flow rate was varied. This resulted in a series of coatings as shown in Table 1. Before the main W-S-N coating, a Cr interlayer and CrN-WS_2_ gradient layer were also deposited to enhance the adhesion of the coatings to the substrates. Fecralloy^®^ substrates were used in this study. Fecralloy^®^ is a registered trademark being an iron-based alloy with chromium, aluminium and yttrium additions (Fe72.8/Cr22/Al5/Y 0.1/Zr 0.1). It is structurally thermal stable at very high temperatures having been used as refractory material up to temperatures of 1400 °C. These substrates were first ground using emery papers (grit sizes = P180 to P1200) and then fine-polished using diamond suspension (size = 3 μm) to achieve a final roughness (Ra) less than ~0.05 μm. The substrates were cleaned in acetone and ethanol for 15 min each, before placing them in the chamber. The rotating sample holder (rotation speed = 17 rotations/min) was fixed at a distance of 100 mm from each target. After placing the substrates, the deposition chamber was pumped down to a base pressure of ~1 × 10^−5^ Pa. This was followed by substrate etching by pulsed DC bias for 40 min and target cleaning for 20 min in Ar gas atmosphere under a pressure of 0.3 Pa. The cleaning parameters were: 500 W for Cr and WS_2_ targets, 240 V for the substrates. After that, the interlayer of Cr and gradient layers of CrN and CrN/WS_2_ were deposited under a pressure of 0.53 Pa. Then, the deposition of the final W-S-N coating was carried out. For this, the power applied to the WS_2_ target was fixed at 350 W, while the N flow rate was varied to obtain different compositions. The total final pressure was fixed at 0.3 Pa while a total deposition time of 120 min was used for all the depositions. A total of 4 coatings with N flow rates of 0, 5, 12.5 and 20 sccm were used in this study (see Table 1). Further details of the deposition process are available in the reference [37].

### 2.2. Characterisation of the As-Deposited Coatings

Wavelength-dispersive spectroscopy (WDS-Oxford Instruments, Abingdon, UK) operated through INCA software under 15 kV accelerating voltage was used for the measurement of the chemical composition of the coatings. Scanning electron microscopy (FESEM-Zeiss Merlin, Oberkochen, Germany) was used to study the surface morphologies of the coatings. X-ray diffraction (XRD-X’ Pert Pro MPD diffractometer, Almelo, The Netherlands) was performed in grazing (3°) mode using copper K_α1_ (λ = 1.5406 Å) source. Nanoindentation (Micro Materials Nano Test platform), with a Berkovich diamond indenter, was used for the analysis of the hardness of the coatings using the method developed by Oliver and Pharr [38]. The applied load (2 mN) was selected after some preliminary tests using dissimilar loads with the aim to use a load so that the total indentation depth was kept to less than 10% of the final coating thickness. For each sample, measurements were taken from two different zones. A total of 16 measurements were acquired at each zone; then, the average was calculated.

### 2.3. Annealing of the Coatings

Annealing of the as-deposited coatings at two different temperatures, 200 °C and 400 °C, was performed in a tube furnace (Adamel—Lhomargy Division D Instruments S.A, Vitry-sur-Seine, France) under protective atmosphere (Ar + 5% vol of H_2_) to study the effect of temperature on the properties of the coatings. After sample placement, the tubular chamber was pumped down to a base pressure of ~5 × 10^−5^ Pa. Then, the protective gas was introduced to obtain a pressure of 0.5 Pa. A ramping rate of 20 °C/min and a holding time of 3 h were selected for each temperature. After annealing, the coatings were characterised using similar techniques described for the as-deposited coatings.

## 3. Results and Discussion

### 3.1. Chemical Composition of as-Deposited and Annealed Coatings

Table 2 shows the chemical composition of the as-deposited coatings and after annealing at 200 °C and 400 °C. The increments of N flow from 0 sccm to 20 sccm led to an increase in the N content from 0 to 25.5 at. % in the coatings. There is no linear/direct relation between the N-flow in the chamber and N content in the coatings. However, all the as-deposited coatings are sub-stoichiometric with respect to S (in the WS_2_ compound).

In general, the sub-stoichiometry with respect to S is mainly due to the atomic mass difference between W and S, the reflected Ar neutrals and the negative self-biasing developed at the substrates during sputtering. All these factors can be responsible for the preferential resputtering of the lighter S element from the growing coating with respect to W. The resputtering of the lighter element due to the reflected Ar neutrals and self-biasing is a well-known phenomenon in the literature for the TMD coatings [31,39,40]. The reasons behind these properties were explained in detail by the current authors in reference [37].

Annealing of the coatings at either 200 °C or 400 °C does not affect the chemical composition significantly as it almost remains constant. In few cases, very small variations were detected but they lie in the range of the error of the measuring equipment. This consistency of the compositional results shows that high temperature does not affect the composition of the coatings.

### 3.2. Morphology of as-Deposited and Annealed Coatings

**Figure 1:**
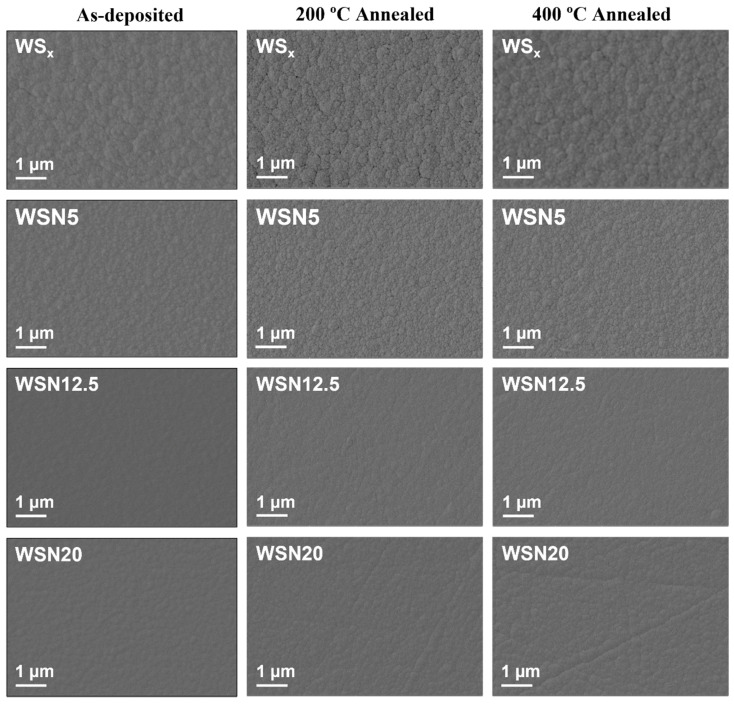
Scanning electron microscopy micrographs showing the surface morphologies of the as-deposited and annealed coatings.

The surface morphology of the as-deposited coatings is shown in Figure 1. The WS_x_ coating does not display any sponge-like and highly porous surface morphology, unlike the ones reported in the literature for pure sputtered TMD coatings [15,26,28]. The deviation from what is most reported in the literature is attributed to the low S/W ratio of the pure coating, which makes the coatings more compact with much less porosity. The coating shows cauliflower-like surface features. These features are typical of surface-limited growing conditions [7,41]. With the addition of N, the compactness of the coatings is increased. The WSN12.5 and WSN20 coatings show the highest compactness. Due to this increment of the compactness, fewer surface features are visible in the micrographs of the WSN12.5 and WSN20 coatings. For a deeper understanding of the compactness versus N content relation and the reasons governing these features, the previous study, i.e., reference [37], provides good insight.

Annealing of the as-deposited coatings at 200 °C and 400 °C does not affect the surface morphology and the results are almost similar to the as-deposited coatings. It should be noted that we have noticed this observation for many compact coatings, unlike what is usually reported in the literature.

### 3.3. Structural Analysis of as-Deposited and Annealed Coatings

**Figure 2:**
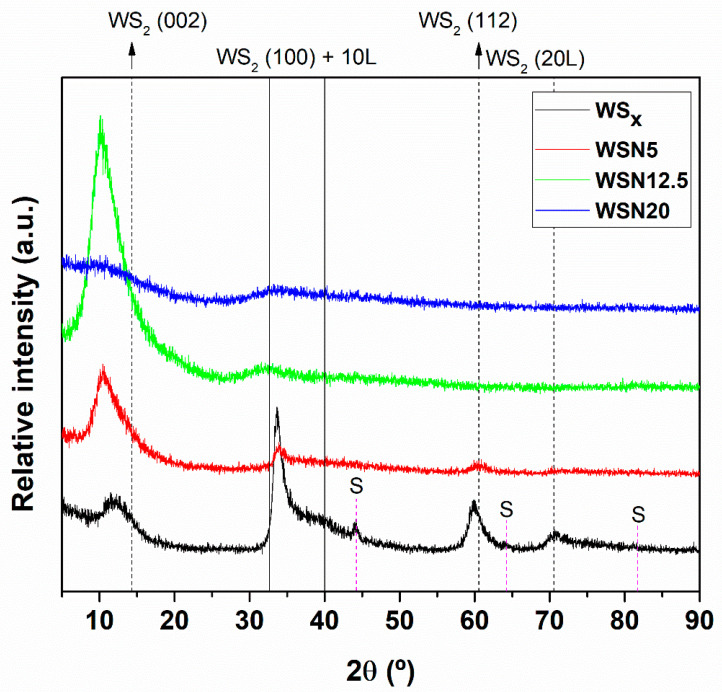
X-ray diffraction patterns of the as-deposited coatings (WS_2_ ICCD card: 087-2417).

The structural analysis results of as-deposited coatings obtained using X-ray diffraction analysis are shown in Figure 2. The WS_x_ pure coating shows X-ray diffraction patterns of typical sputtered TMD coatings. This coating displays a (100) preferential orientation with the most prominent peaks observed at ~30–45° being related to the (100) and (10 L) planes (where L = 1, 2, 3, …). The other major peak from the XRD diffractogram is the (002) located at ~12°. This peak shifts to the left when compared to the pure WS_2_ standard (ICCD card: 087-2417). The shift to lower angles is attributed to the lower S/W ratio with respect to WS_2_ and, probably, some additional contribution from the residual stresses. The peaks at ~62° and ~71° are related to the (112) and (20 L) planes of WS_2_. Diffraction peaks from the substrate are also highlighted in Figure 2. With N introduction in the coatings, the preferential orientation shifts to (002). The WSN5 coating with low N-alloying displays a broad but high-intensity (002) peak and a lower-intensity (100) peak. With the further increase in N, the WSN12.5 coating has the highest-intensity (002) peak in comparison to the other coatings. This coating does not show any vestiges of the (100) peak. Finally, the coating with the highest N content, WSN20, is amorphous without any defined (002) or (100) peaks. This is probably due to the fact that the N-alloying of 25.5 at. % completely disturbs the structural growth of TMD crystals. There is a very high possibility that very small nanocrystals of WS_2_ are present, but they are smaller in size than the detection limit of the XRD (i.e., less than 5 nm). Overall, in all the N-alloyed coatings, the (002) peak shifted to lower angles, which is due to the combined effects of: (i) low stoichiometry of S, (ii) incorporation of N and (iii) induced residual stresses [42,43,44]. Further information about the reason for the above-mentioned properties can be found in reference [37].

**Figure 3:**
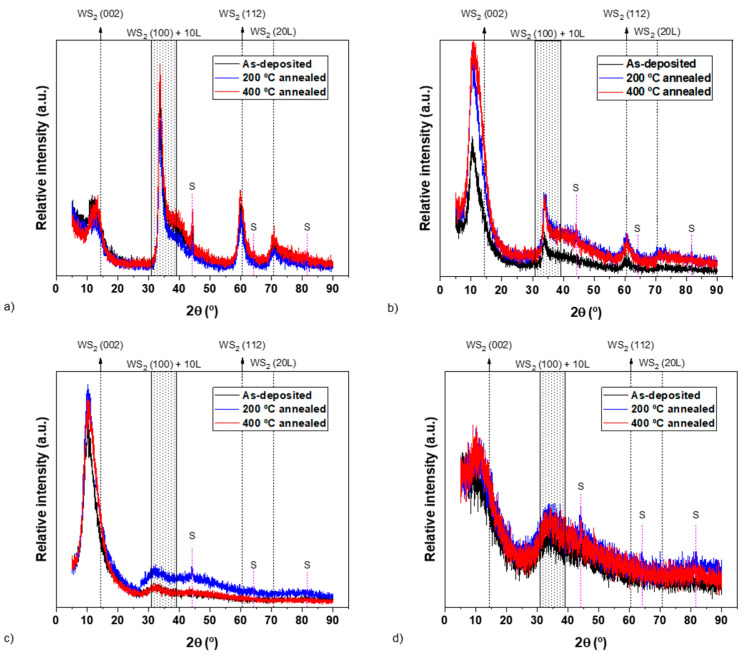
X-ray diffraction patterns of the as-deposited and annealed coatings (WS_2_ ICCD card: 087-2417)—(**a**) WS_x_, (**b**) WSN5, (**c**) WSN12.5, (**d**) WSN20.

Unlike compositional and morphological observations, annealing marginally influences the crystal structure of the as-deposited coatings, as shown in Figure 3. The intensity of the XRD peaks of annealed WS_x_, WSN5 and WSN12.5 coatings at 200 °C increases as compared to the as-deposited coatings. When the annealing temperature is increased, the increment in the intensity further enhances in WS_x_ and WSN5 coatings, while it remains almost similar to 200 °C annealing for the WSN12.5 coating. The WSN20 amorphous coating also displays a slight increase in the crystallinity at both 200 °C and 400 °C but the increment is marginal.

Another important observation from the annealed XRD results is the slight shift in the (002) peaks to lower angles in the WS_x_ and WSN5 coatings. Considering that no changes in the composition with annealing could be observed, this may be directly linked to the release of residual stresses. This shift corroborates our previous claim that some residual stresses might be contributing to the shift in (002) peaks to lower diffraction angles.

### 3.4. Hardness of as-Deposited and Annealed Coatings

**Figure 4:**
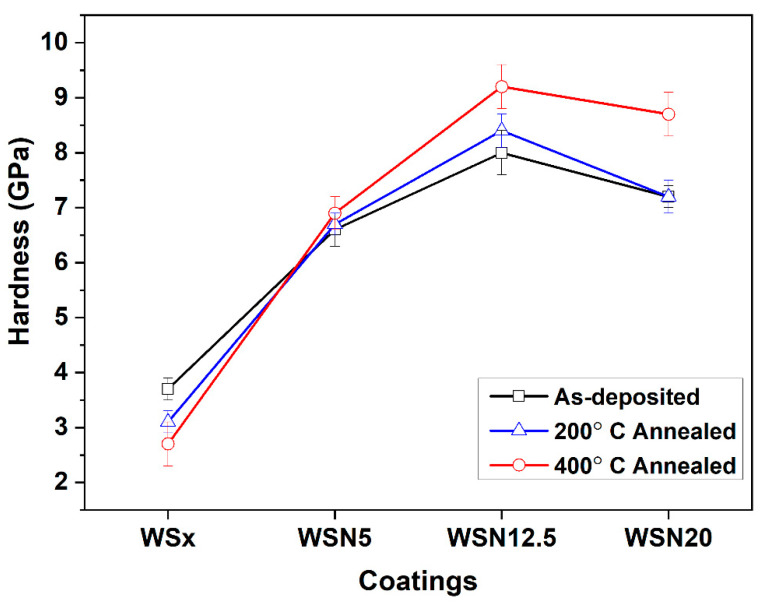
Hardness of the as-deposited and annealed coatings.

Figure 4 shows the hardness measurements of the as-deposited and annealed coatings. For the as-deposited coatings, the hardness of the WS_x_ coating is the lowest (3.7 GPa) while the WSN12.5 coating displays the maximum hardness (8 GPa). Overall, the addition of N enhanced the hardness of the coatings. Firstly, the lower hardness of WS_x_ coating is due to its comparatively higher porosity as compared to the N-alloyed coatings. However, this value is higher than those typically reported for pure sputtered WS_2_ in the literature [45,46,47]. Compared to the ones mentioned in the literature, this difference can be attributed to a non-columnar morphology, a higher compactness and a lower S/W ratio (Ref. [37] shows the cross-sectional morphology of these coatings along with a detailed discussion). Moreover, as is discussed above, the addition of N makes the coatings more compact, justifying their higher hardness than the WS_x_. Changes in the chemical bonding of coatings with N incorporation may also account for the hardness increase. Finally, the highest value shown by WSN12.5 is related to the crystal structure of this coating. As reported by Mutafov et al. [44], the coatings with (002) planes preferentially oriented parallel to the surface appear harder in nanoindentation tests since they resist better to the penetration of the indenter. The decrease in the hardness of the WSN20 coating is due to the loss of crystallinity, as previously shown in Figure 2.

Although no significant changes in the hardness values could be observed after annealing of the samples in relation to as-deposited conditions, there is clearly a trend that starts to be observed after 200 °C annealing and is enhanced after 400 °C, as follows: for the N-unalloyed coating, the annealing led to a decrease in the hardness after annealing, whereas an inverse evolution is observed for N-containing coatings. If this improvement in the hardness can be understood for the WSN20 coating based on an increase in the crystallinity, as shown in Figure 3d, such an explanation cannot be withstood for the other samples for which there is no structural transformation. This inverse trend can only be explained by the change in the preferential orientation when N is incorporated in the coatings (see Figure 2). In fact, a progressive increase in the (002) intensity is observed with increasing N content. How can this change have an impact on the hardness after annealing? During cooling down from the annealing temperature, thermal stresses are created in the coating due to the difference in the thermal expansion coefficient (TEC) between the coating and the substrate. These stresses will be of tensile or compressive types if the TEC of the substrate is lower or higher that the TEC of the coating, respectively. It is well known that TMDs have extreme anisotropic thermal and electrical properties following the orientation of the structure, i.e., there is a big difference in TEC values between a and c axes, being much higher in the c direction [48,49]. No precise values are found in the literature for sputtered W-S films; however, the values for WS_2_ crystals are close to the ones of the Fecralloy^®^ substrate, making it possible to speculate that the one from the substrate can be in the middle of those from the c and a axis of WS_2_. Therefore, for the WS_x_ coating, which has a (100) orientation (and the (002) planes perpendicular to the surface-c direction parallel to the substrate), when exposed to high temperature and then cooled down, the difference between the TEC of the film and substrate leads to tensile stresses and, consequently, to a decrease in the hardness values. In the case of the N-rich coatings, which have a (002) preferential orientation and, consequently, the (002) planes are parallel to the surface, when cooling down from high temperature, compressive stresses will be created. This phenomenon leads to an increase in hardness values for the N-rich coatings. The higher the degree of (002) orientation, the higher the hardness difference between the room-temperature and high-temperature-annealed coatings.

The hardness of the as-deposited coatings slightly changed after annealing. Since no chemical composition variations are promoted by annealing, the hardness response can be interpreted based on the combined effects of: (i) changes in crystal structure, as presented in Figure 3, (ii) differences in thermal expansion coefficient (TEC) between the a and c axis of the W-S-N coatings and (iii) different thermal expansion coefficient between the coating and the substrate. It is well known that TMDs have quite big differences in TEC values between a and c axes [48,49]. Thus, for the WS_x_ coating which has a (100) orientation (and the (002) planes perpendicular to the surface), when exposed to high temperature and then cooled down, the difference between the TEC of the film and substrate causes the morphology to open more, leading to a decrease in the hardness values. Although an increase in crystallinity is observed for the WS_x_ coating with temperature, this is not enough to counteract the influence of the decrease in hardness promoted by the above-mentioned effect. Similarly, in the case of the N-rich coatings, which have a (002) preferential orientation and, consequently, the (002) planes are parallel to the surface, when cooling down from high temperature, the morphology will close more. This phenomenon coupled with the increase in crystallinity of the coatings leads to an increase in hardness values for the N-rich coatings. The higher the degree of (002) orientation, the higher the hardness difference will be between the room-temperature and high-temperature annealed coatings.

## 4. Conclusions

In this study, the impact of annealing on the composition, morphology, structure and hardness of W-S-N coatings is investigated. For this purpose, a set of W-S-N coatings with increasing N concentration from 0 up to 25.5 at. % were deposited using DC reactive magnetron sputtering. The as-deposited coatings are sub-stoichiometric with respect to S in WS_2_. The morphologies of N-alloyed coatings were more compact than the pure coating, WS_x_. In the as-deposited coatings, the highest hardness achieved was 8.0 GPa. Annealing at 200 °C and 400 °C did not influence the composition and morphology of the coatings. Only slight variations in the structure and hardness of the coatings were detected. After annealing, the highest hardness detected was 9.2 GPa for the WSN12.5 coating. The hardness variations with annealing are justified by the combined effects of the changes in crystal structure, the differences in thermal expansion coefficient between the a and c axis of the coatings and the different thermal expansion coefficient between the coatings and the substrate. It can be deduced from the hardness results of the N-rich coatings that the higher the difference between the intensity of the (002) diffraction peak of the as-deposited and annealed coatings, the higher the difference in hardness.

## Figures and Tables

**Table 1:** Main deposition parameters used during the synthesis of the coatings and the achieved thickness.

Coating Designation	WS_2_ Target Power (W)	N Flow Rate (Sccm)	Total Deposition Time (Min)	Outer W-S-N Coating Thickness (µm)
	350	0	120	1.12
WSN5	350	5	120	1.21
WSN12.5	350	12.5	120	1.16
WSN20	350	20	120	1.10

**Table 2:** Chemical composition of the as-deposited and annealed coatings.

Coatings	N (at. %)	W (at. %)	S (at. %)	S/W
**As-deposited condition**				
**WS_x_**	-	40.0 ± 1.5	59.0 ± 1.2	1.47
**WSN5**	14.6 ± 0.5	39.0 ± 0.1	45.6 ± 0.5	1.17
**WSN12.5**	23.0 ± 1.8	38.1 ± 0.9	37.9 ± 0.8	0.99
**WSN20**	25.5 ± 0.2	35.6 ± 0.4	38.1 ± 0.2	1.07
**After 200 °C annealing**				
**WS_x_**	-	40.7 ± 0.3	59.3 ± 0.5	1.46
**WSN5**	14.2 ± 0.2	38.8 ± 0.4	47.0 ± 0.2	1.21
**WSN12.5**	21.2 ± 0.8	39.1 ± 1.0	39.4 ± 0.7	1.00
**WSN20**	25.3 ± 0.3	35.1 ± 0.7	39.7 ± 1.1	1.11
**After 400 °C annealing**				
**WS_x_**	-	40.4 ± 0.3	59.6 ± 0.6	1.48
**WSN5**	13.6 ± 0.4	39.9 ± 0.9	46.6 ± 0.8	1.17
**WSN12.5**	20.5 ± 0.9	39.5 ± 0.4	40.0 ± 0.3	1.00
**WSN20**	24.6 ± 0.4	35.8 ± 0.7	39.6 ± 0.2	1.11

## Data Availability

Not applicable.
